# Increased Expression of Meteorin-Like Hormone in Type 2 Diabetes and Obesity and Its Association with Irisin

**DOI:** 10.3390/cells8101283

**Published:** 2019-10-19

**Authors:** Irina AlKhairi, Preethi Cherian, Mohamed Abu-Farha, Ashraf Al Madhoun, Rasheeba Nizam, Motasem Melhem, Mohamed Jamal, Suleiman Al-Sabah, Hamad Ali, Jaakko Tuomilehto, Fahd Al-Mulla, Jehad Abubaker

**Affiliations:** 1 Department of Biochemistry and Molecular Biology, Dasman Diabetes Institute, Kuwait City 15462, Kuwait; irina.alkhairi@dasmaninstitute.org (I.A.); preethi.cherian@dasmaninstitute.org (P.C.); 2 Department of Genetic and Bioinformatics, Dasman Diabetes Institute, Kuwait City 15462, Kuwait; ashraf.madhoun@dasmaninstitute.org (A.A.M.); rasheeba.iqbal@dasmaninstitute.org (R.N.); motasem.melhem@dasmaninstitute.org (M.M.); hamad.ali@HSC.EDU.KW (H.A.); 3 Department of Surgery, Faculty of Medicine, Health Sciences Centre, Kuwait University, Sulaibekhat 90805, Kuwait; mjamal110@gmail.com; 4 Department of Pharmacology & Toxicology, Faculty of Medicine, Health Sciences Centre, Kuwait University, Sulaibekhat 90805, Kuwait; suleiman@HSC.EDU.KW; 5 Department of Medical Laboratory Sciences, Faculty of Allied Health Sciences, Health Sciences Centre, Kuwait University, Sulaibekhat 90805, Kuwait; 6 Research Division, Dasman Diabetes Institute, Kuwait City 15462, Kuwait; tuomilehto@hotmail.com

**Keywords:** Meteorin-like hormone, METRNL, irisin, adipomyokines, type 2 diabetes, obesity

## Abstract

Type 2 diabetes (T2D) is a growing pandemic associated with metabolic dysregulation and chronic inflammation. Meteorin-like hormone (METRNL) is an adipomyokine that is linked to T2D. Our objective was to evaluate the changes in METRNL levels in T2D and obesity and assess the association of METRNL levels with irisin. Overall, 228 Arab individuals were enrolled. Plasma levels of METRNL and irisin were assessed using immunoassay. Plasma levels of METRNL and irisin were significantly higher in T2D patients than in non-diabetic patients (*p* < 0.05). When the population was stratified based on obesity, METRNL and irisin levels were significantly higher in obese than in non-obese individuals (*p* < 0.05). We found a significant positive correlation between METRNL and irisin (r = 0.233 and *p* = 0.001). Additionally, METRNL and irisin showed significant correlation with various metabolic biomarkers associated with T2D and Obesity. Our data shows elevated METRNL plasma levels in individuals with T2D, further exacerbated with obesity. Additionally, a strong positive association was observed between METRNL and irisin. Further studies are necessary to examine the role of these proteins in T2D and obesity, against their ethnic background and to understand the mechanistic significance of their possible interplay.

## 1. Introduction

Type 2 diabetes (T2D) is a leading global cause of early mortality and morbidity, and is associated with numerous macrovascular and microvascular complications including retinopathy, nephropathy, neuropathy, and cardiovascular disease [1]. T2D is mainly characterized by relative insulin deficiency due to pancreatic β-cell dysfunction and insulin resistance in target organs [2]. Molecular and phenotypic changes in adipose tissue, skeletal muscle, and the liver are involved in the development of insulin resistance and eventually T2D [3]. The skeletal muscle accounts for approximately 75% of whole-body insulin-stimulated glucose uptake; hence tissue defects play a critical role in glucose homeostasis in T2D patients [3]. Insulin resistance partly develops due to impaired mitochondrial biogenesis [4] and accumulation of toxic lipid metabolites such as diacylglycerols and ceramides in skeletal muscle [5]. Skeletal muscle was recently shown to have endocrine properties, with muscle cells secreting intrinsically active metabolites known as myokines [6,7]. These signaling peptides are released in response to several stimuli, including muscle contraction during physical activity and/or nutritional changes [8]. Myokines mediate muscle growth (myogenesis) and regeneration within the muscle itself and are a part of a complex network that mediates crosstalk between muscles, liver, adipose tissue, the brain, and other organs [9]. Thus myokines are involved in modulating body metabolism. Interleukin-6 (IL-6), brain-derived neurotrophic factor (BDNF) and interleukin-15 (IL-15) are known myokines that were shown to increase muscle mass and increase fatty acid oxidation [10].

Peroxisome proliferator-activated receptor (PPAR)-γ coactivator (PGC-1α) is a transcription coactivator that plays a vital role in regulating cellular energy metabolism. It acts as a molecular switch for multiple cellular processes, including mitochondrial biogenesis and respiration, gluconeogenesis and glucose transport, glycogenolysis, fatty acid oxidation, peroxisomal remodeling, muscle fiber-type switching, and oxidative phosphorylation [11]. Thus, making PGC-1α and the factors regulated by this protein valuable targets in the study of diabetes and obesity. It is reported that PGC-1α expression is downregulated in people with T2D [12]. Numerous studies have established a causal relationship between PGC-1α dysregulation in skeletal muscle and abnormal energy homeostasis as well as between insulin resistance and T2D [13]. In addition, PGC-1α expression level increases in skeletal muscles with exercise, which in turn increases exercise capacity, induces angiogenesis, and prevents muscle atrophy and degeneration [14,15].

Several gain- and loss-of-function models have been established based on the importance of PGC-1α in skeletal muscle energy homeostasis and morphogenesis. Irisin [16], β-aminoisobutyric acid (BAIBA) [17] and Meteorin-like (METRNL) [18] are novel myokines that were recently identified through PGC-1α overexpression in muscle. Interestingly, all three myokines appear to be involved in the promotion of beige fat thermogenesis. Beige fat are thermogenic adipocytes found to be distributed within the white adipose tissue. Like the brown fat, they release heat by oxidation of fatty acids upon stimulation, have increased mitochondrial density and have the capacity for uncoupled respiration, making them important promoters of metabolic homeostasis [19].

Irisin is a proteolytic derivative of the muscle fibronectin type III domain-containing protein 5 (FNDC5) that is released into the bloodstream. An interesting aspect of this protein is that the amino acid sequence is identical among mammalian species, suggesting that its function is highly conserved [20]. Spiegelman’s group first reported that exercise increases FNDC5 expression in muscle, which is cleaved and secreted as the hormone irisin [16]. Moreover, they reported that irisin is upregulated by PGC-1α, initiating a thermogenic program in white adipose tissue by activating uncoupling protein 1 (UCP1). Other studies have demonstrated that irisin is not only present in muscle tissue but also is produced by adipocytes [21,22]. Furthermore, FNDC5 was overexpressed using lentiviral injection or with persistent subcutaneous perfusion of irisin. The increased level of FNDC5/irisin led to increased energy expenditure, improved glucose/lipid metabolic derangements, and attenuated insulin resistance in high-fat diet-induced obese mice [23]. Together, these studies add value to the potential of FNDC5/irisin as an effective strategy in attenuating metabolic dysregulation and insulin resistance in obesity and T2D.

Another novel hormone identified by PGC-1α overexpression in skeletal muscles is the METRNL hormone. Rao et al. showed that the mRNA level of METRNL increased with increasing PGC-1α level, and that exercise and cold exposure stimulate METRNL expression in adipose tissue and increase its level in circulation. They reported that METRNL recruits eosinophils into the adipose tissue, which are the major source of the cytokines IL-4 and IL-13. These immune cytokines are known to induce the expression of thermogenic genes, suggesting that this hormone is involved in thermogenesis and plays a role in modulating energy expenditure [18]. Hence, METRNL an interesting target to investigate in relation to metabolic disorders such as obesity and diabetes.

We now know that both adipomyokines METRNL and irisin are regulated by the transcription factor PGC-1α. Also, both these adipomyokines are involved in stimulating white adipose tissue browning and promote glucose uptake in the skeletal muscle and heart. Therefore, we were interested to investigate the expression of these molecules in Arab individuals with obesity and T2D. We also wanted to understand if there is any association between these two adipomyokines and study their correlation with other biochemical markers associated with metabolic dysregulation.

## 2. Materials and Methods

### 2.1. Study Population

This study was conducted on 228 Arab adult men and women with and without T2D (124 non-diabetic [73 non-obese and 51 obese] and 104 T2D [38 non-obese and 66 obese]) individuals. T2D was defined by fasting plasma glucose ≥ 126 mg/L (7 mmol/L), under treatment, or self-reports of previously diagnosed T2D [24,25,26]. Obesity was defined based on body mass index (BMI), where participants with a BMI > 30 kg/m^2^ were considered obese and those with a BMI between 20 and 30 kg/m^2^ were considered non-obese. All participants provided written informed consent prior to participating in the study. The study was approved by the ethical review board of Dasman Diabetes Institute (Project#: RA-2016-045) and has been conducted in accordance with the ethical guidelines of the Declaration of Helsinki. Morbidly obese patients (BMI > 40 kg/m^2^) with prior major illness and/or who were taking medication or supplements known to influence body composition or bone mass were excluded from the study.

### 2.2. Blood and Tissue Sampling

Venous blood samples were collected using Vacutainer Ethylenediaminetetraacetic Acid (EDTA) tubes after fasting for a minimum of 8 h. The blood was centrifuged at 400× *g* for 10 min at room temperature. The plasma was separated, aliquoted, and stored at −80 °C until the assay was performed.

### 2.3. Anthropometric Measurements and Blood Biochemistry

Anthropometric measurements of height and weight were obtained to calculate the BMI using a ratio of weight (kg) to height in meters squared (m). Fasting blood glucose (FBG) and lipid profiles including triglycerides (TG), low-density lipoprotein (LDL), and high-density lipoprotein (HDL), as well as total cholesterol (TC) were measured using a Siemens Dimension RxL chemistry analyzer (Diamond Diagnostics, Holliston, MA, USA). Glycated hemoglobin (HbA1c) levels were determined using the VariantTM device (Bio-Rad, Hercules, CA, USA). Insulin and C-peptide plasma levels were measured using the enzyme-linked immunosorbent assay (ELISA) (Mercodia Developing Diagnostics, Ltd., Uppsala, Sweden, Cat #10-1136-01 for C-peptide and #10-1113-01 for insulin). All of the assays described above were performed according to the manufacturer’s instructions. Insulin resistance was calculated using the Homeostatic Model Assessment for Insulin Resistance HOMA-IR formula: FBG (mg/L) × fasting insulin (mU/L)/22.5. Furthermore, the homeostatic model assessment (HOMA) method was used to assess β-cell function (HOMA-β): HOMA-β% = [20 × fasting insulin (mU/L)]/glucose (mg/L) − 3.5 × 100 [27].

### 2.4. Measurement of Plasma Levels of METRNL and Irisin Using ELISA

Plasma levels of METRNL and irisin were detected using an ELISA assay. Plasma samples were thawed on ice and centrifuged for 5 min at 10,000× *g* at 4 °C to remove any remaining cells or platelets. The plasma level of METRNL was measured using the human METRNL ELISA kit (LifeSpan BioSciences, Inc., Seattle, WA, USA. Cat# LS-F13315). Samples were diluted 10× with sample diluent. ELISA was performed according to the manufacturer’s instructions. The intra-assay coefficient of variation was 5.0–10.0% while the inter-assay coefficient of variation was <10%. Plasma level of irisin was measured using the irisin recombinant enzyme immunoassay kit (Phoenix Pharmaceuticals, Inc., Burlingame, CA, USA. Cat #EK-067-29) following the protocol in the instructions for the kit. Plasma samples were diluted 40× with the 1× assay buffer (provided in the kit). The intra-assay coefficient for this ELISA assay was 1.0–7.0%, while the inter-assay coefficient was <20%.

### 2.5. Statistics

The Mann–Whitney *U* test was used to compare clinical parameters between T2D and non-diabetic study participants and between non-obese and obese individuals. The Mann–Whitney *U* test was also used to compare the plasma levels of METRNL and irisin. Spearman partial correlation adjusting for age and gender was used to determine the association of METRNL with irisin and to study the correlation of these proteins with clinical and biochemical parameters included in this study. All data were reported as mean ± standard error of mean. Statistical assessment was 2-sided and considered statistically significant at *p* < 0.05. All analyses were performed using SPSS software (SPSS Statistics V24.0, IBM, New York, NY, United States).

## 3. Results

### 3.1. Characteristics of the Study Population

Selected characteristics of our sample population were stratified as T2D and non-diabetic based on their fasting plasma blood glucose values presented in Table 1. Age, BMI, waist/hip ratio, HDL, FBG, HbA1c, and TGL were significantly higher in T2D than in non-diabetic individuals (*p* < 0.05). In addition, insulin and C-peptide plasma levels were significantly increased in patients with T2D when compared to non-diabetic patients, and the HOMA results showed higher values in T2D individuals. Classifying the population set based on obesity, obese individuals showed significantly higher BMI, waist/hip ratio, FBG, and HbA1c (Table 2). Further stratifying the study population into four groups (non-diabetic: non-obese and obese, and T2D: non-obese and obese), the difference in BMI and waist/hip ratio was significantly higher in the non-diabetic obese patients when compared to the non-diabetic non-obese patients (*p* < 0.05). Moreover, the obese group demonstrated a significantly higher value for HOMA-ß. However, the T2D obese group showed significantly higher BMI, FBG, and HbA1c when compared to the T2D non-obese group (Table 3).

### 3.2. METRNL and Irisin Expression in the Circulation

**Figure 1:**
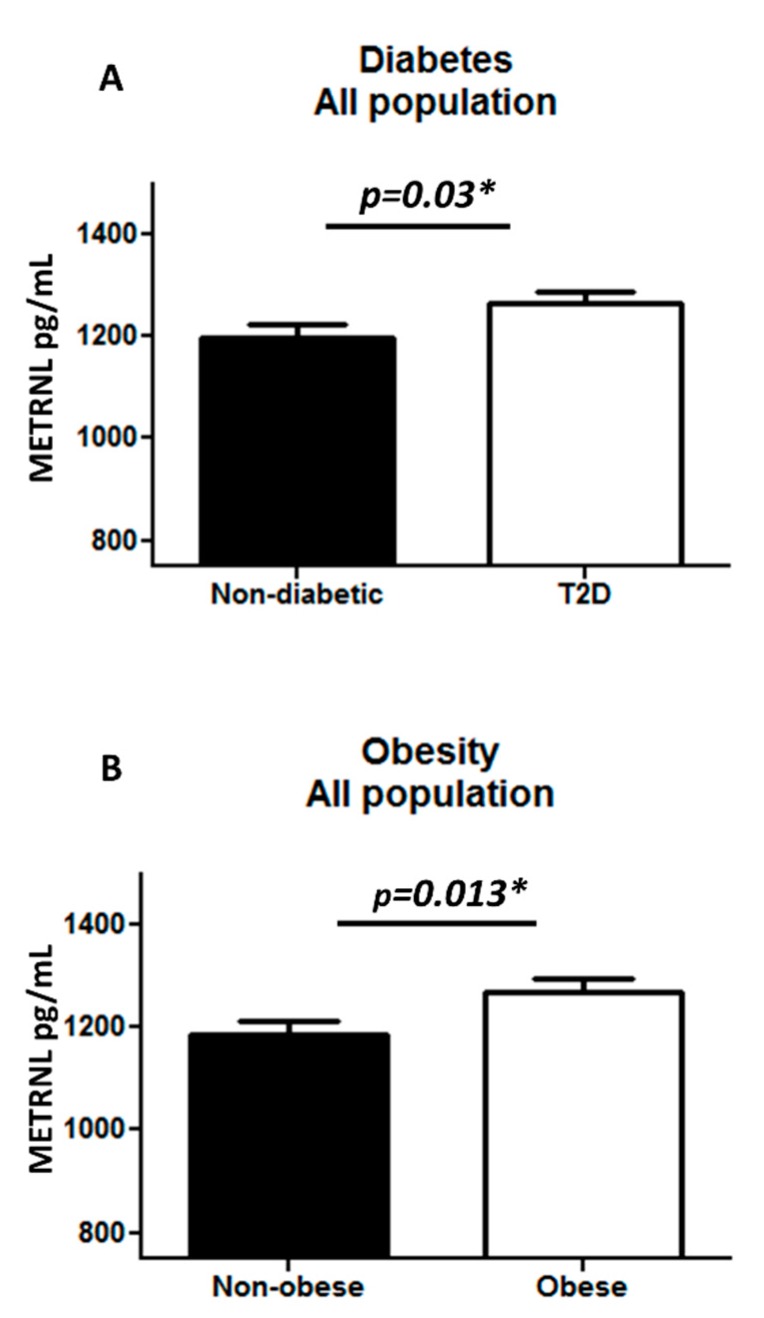
Meteorin-like hormone (METRNL) level in plasma in all populations (n = 228). (**A**) Comparing non-diabetic to T2D individuals; (**B**) comparing non-obese to obese individuals. The METRNL level in plasma was determined using enzyme linked immunosorbent assay (ELISA). The population was classified on the basis of their diabetic status (**A**). Diabetes was defined by fasting plasma glucose ≥ 126 mg/L (7 mmol/L). Furthermore, the population was classified on the basis of obesity (**B**). Obesity was defined based on BMI, where participants with BMI > 30 kg/m^2^ were considered obese and those with BMI between 20 and 30 kg/m^2^ were considered non-obese. Statistical assessment was 2-sided and considered statistically significant at * *p* < 0.05.

**Figure 2:**
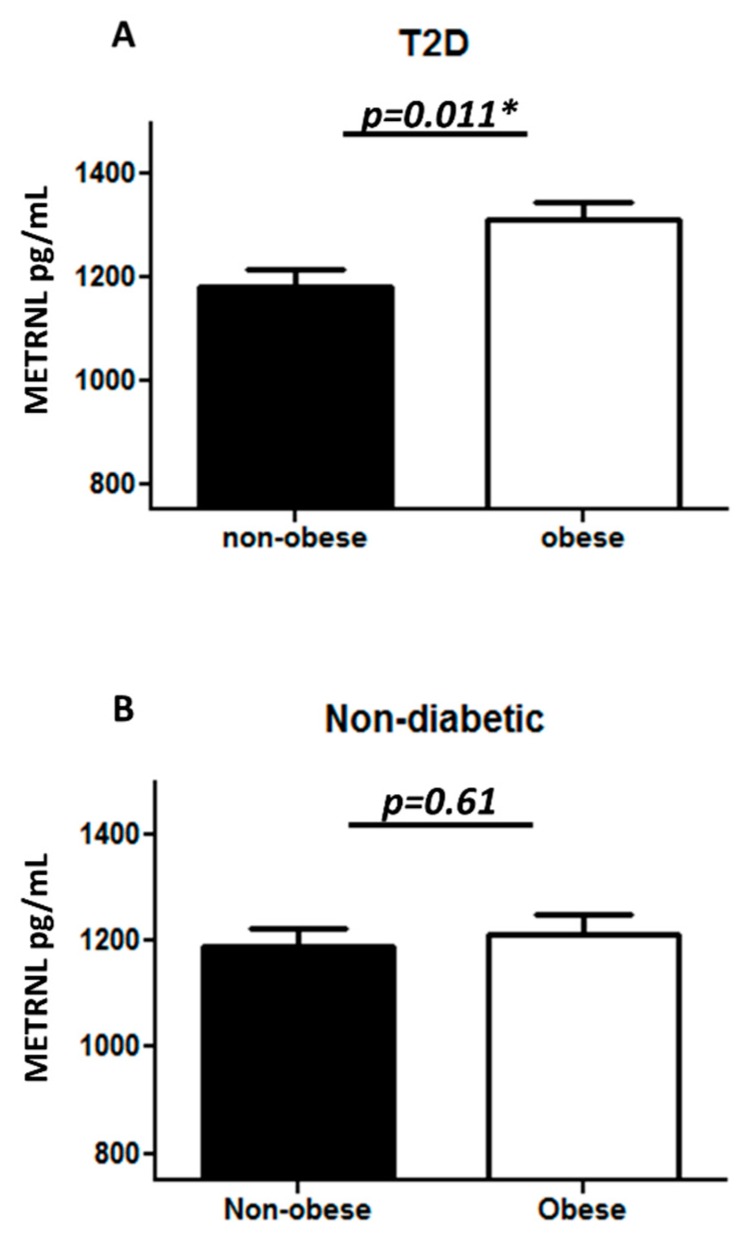
METRNL level in plasma following sub-classification into four groups: non-diabetic non-obese and obese (n = 124), and T2D non-obese and obese (n = 104). (**A**) Comparing non-diabetic non-obese to obese individuals; (**B**) plasma level of METRNL in T2D comparing non-obese to obese individuals. METRNL level in plasma was determined using enzyme linked immunosorbent assay. The population was classified on the basis of their diabetic status and further sub-classified on the basis of obesity. Diabetes was defined by fasting plasma glucose ≥ 126 mg/L (7 mmol/L). Obesity was defined based on BMI, where participants with BMI > 30 kg/m^2^ were considered obese and those with BMI between 20 and 30 kg/m^2^ were considered non-obese. Statistical assessment was 2-sided and considered statistically significant at * *p* < 0.05.

The plasma level of METRNL was significantly higher in T2D individuals (1263.52 ± 24.97 pg/mL) as compared to non-diabetic individuals (1198.58 ± 24.28 pg/mL) with *p* = 0.03 (Figure 1A). Similarly, a significant increase in METRNL level was observed in obese (1267.91 ± 24.83 pg/mL) as compared to non-obese individuals (1186.35 ± 24.22 pg/mL) with *p* = 0.013 (Figure 1B). After further classifying the population into four groups, obese T2D individuals had a significantly higher level of plasma METRNL (1311.88 ± 32.09 pg/mL) when compared to non-obese T2D individuals (1179.52 ± 36.14 pg/mL) (Figure 2A), and no significant difference was observed between non-diabetic obese and non-obese individuals (Figure 2B). Afer further categorization of the samples as lean (BMI < 25), overweight (25 < BMI < 29.9), and obese (BMI > 30), there was a significant increase in METRNL only in T2D obese individuals (Supplementary Figure S1A,B). When the samples were classified based on mean age (46 years) of the population, there was no change in plasma METRNL level with age (Supplementary Figure S2A).

**Figure 3:**
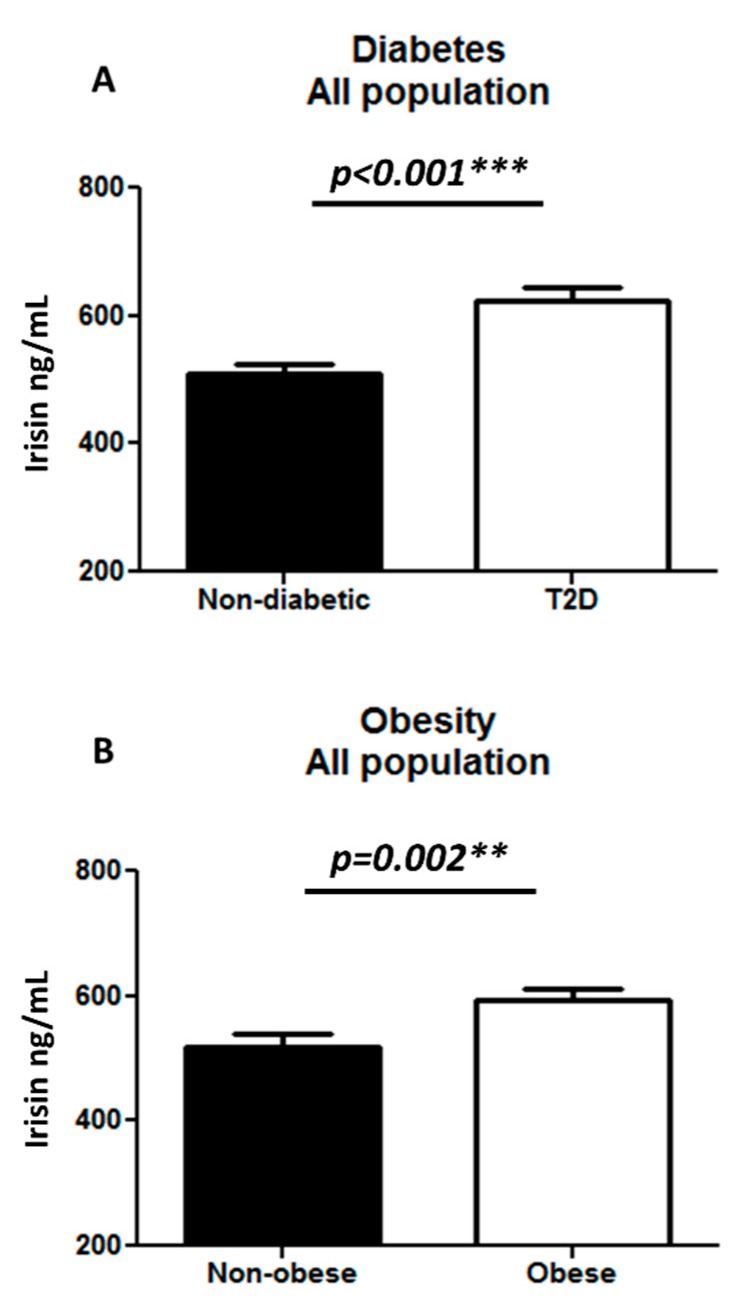
Irisin level in plasma in all populations (n = 228). (**A**) Comparing non-diabetic to T2D individuals; (**B**) comparing non-obese to obese individuals (n = 228). Irisin level in plasma was determined using enzyme linked immunosorbent assay. The population was classified on the basis of their diabetic status (**A**). Diabetes was defined by fasting plasma glucose ≥ 126 mg/L (7 mmol/L). Furthermore, the population was classified on the basis of obesity (**B**). Obesity was defined based on BMI, where participants with BMI > 30 kg/m^2^ were considered obese and those with BMI between 20 and 30 kg/m^2^ were considered non-obese. Statistical assessment was 2-sided and considered statistically significant at ** *p* < 0.01; *** *p* < 0.001.

**Figure 4:**
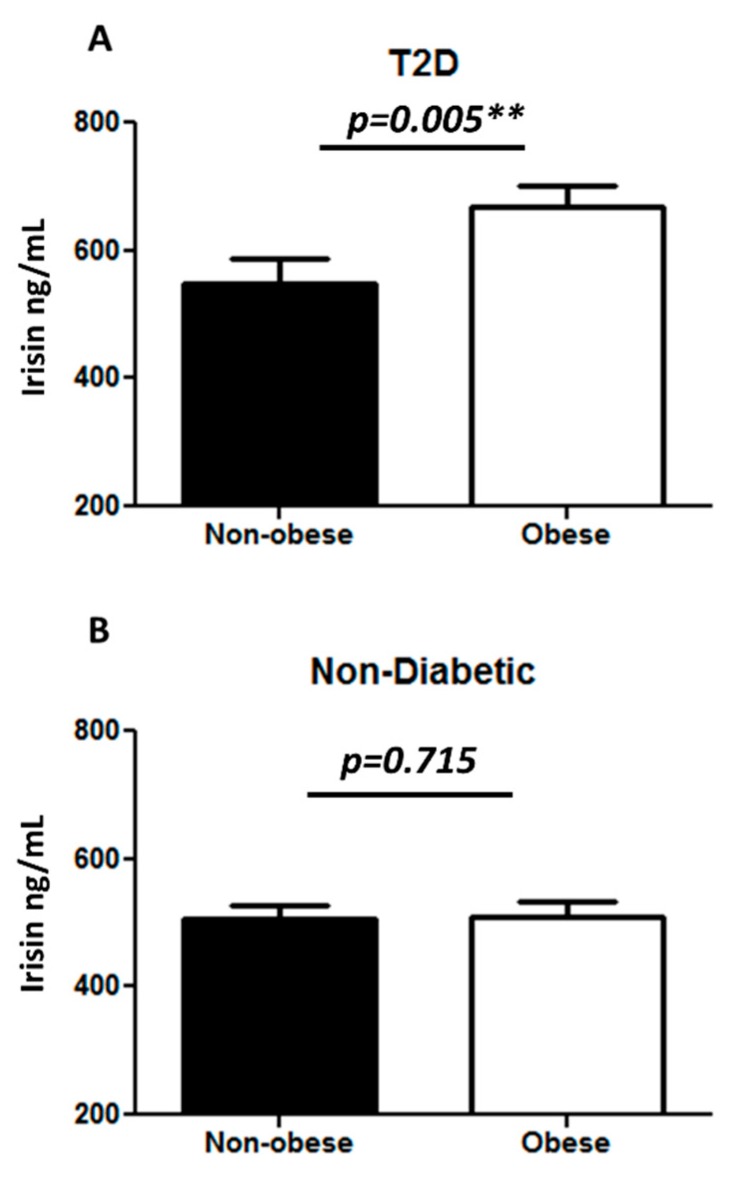
Irisin level in plasma following sub-classification into four groups: non-diabetic non-obese and obese (n = 124) and T2D non-obese and obese (n = 104). (**A**) Comparing the plasma level of irisin in non-diabetic non-obese and obese individuals; (**B**) comparing the plasma level of irisin in T2D non-obese and obese individuals. Irisin level in plasma was determined using enzyme linked immunosorbent assay. The population was classified on the basis of their diabetic status and further sub-classified on the basis of obesity. Diabetes was defined by fasting plasma glucose ≥ 126 mg/L (7 mmol/L). Obesity was defined based on BMI, where participants with BMI > 30 kg/m^2^ were considered obese and those with BMI between 20 and 30 kg/m^2^ were considered non-obese. Statistical assessment was 2-sided and considered statistically significant at ** *p* < 0.01.

In addition, we measured the plasma level of irisin in the study population and found a significantly higher increase in the irisin level in T2D (623.01 ± 21.82 ng/mL) as compared to non-diabetic individuals (508.23 ± 15.38 ng/mL) with *p* < 0.001 (Figure 3A). Classifying the population based on obesity, we observed a significant increase in irisin level in obese (593.42 ± 18.16 ng/mL) as compared to non-obese individuals (519.90 ± 19.42 ng/mL) with *p* = 0.002 (Figure 3B). The plasma level of irisin was significantly increased in the T2D obese (668.05 ± 24.26 ng/mL) as compared to the T2D non-obese individuals (547.08 ± 39.18 ng/mL) with *p* = 0.005 (Figure 4A). No significant difference was observed in the non-diabetic obese and non-obese individuals (Figure 4B).

When the samples were categorized into three groups as lean, overweight and obese to further understand the levels of irisin within each subpopulation, we observed a significant increase in the level of irisin between lean, overweight and obese categories within the T2D population. However, there was only a slight increase in irisin level between lean, overweight and obese within the non-diabetic population. This is data is presented as (Supplementary Figure S3A,B). To understand if age has an effect on the expression level of irisin, we categorized them against the mean age (46 years) of the population under study. We observed a significant increase in the plasma level of irisin with age. This data is presented as (Supplementary Figure S2B).

### 3.3. Correlation Analysis

**Figure 5:**
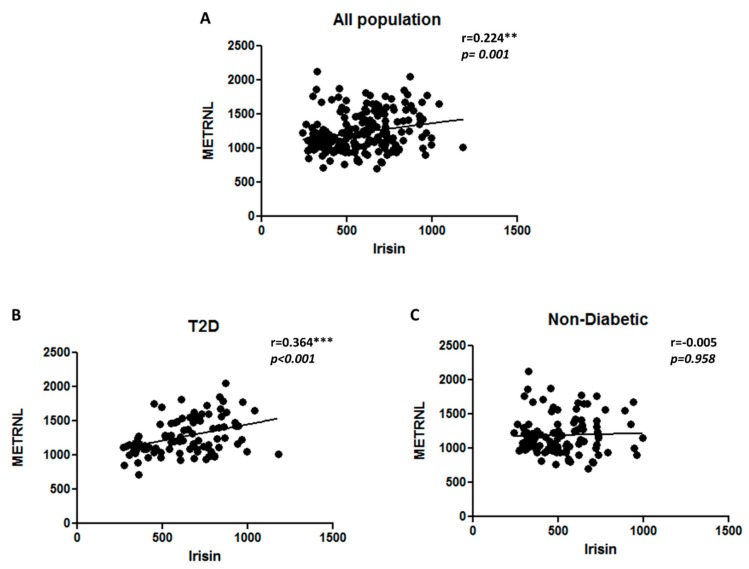
Correlation analysis between METRNL and irisin levels in plasma. (**A**) All populations. (**B**) Individuals with T2D. (**C**) Non-diabetic individuals. METRNL and irisin levels in plasma were determined using enzyme linked immunosorbent assay. Diabetes was defined by fasting plasma glucose ≥ 126 mg/L (7 mmol/L). Spearman correlation coefficient was used to determine the association of METRNL with irisin. Statistical assessment was 2-sided and considered statistically significant at ** *p* < 0.01; *** *p* < 0.001.

Correlation analysis was performed on the entire study population to evaluate the association of METRNL and irisin levels with various clinical parameters and with each other. In order to understand if there are any gender based differences in the correlation results, we performed the correlation analysis categorizing the population based on gender. We observed a significant correlation between METRNL and irisin within the male population. However, this was not seen in the female population. This data is shown in (Supplementary Figure S4A,B). Therefore the results were adjusted for age and gender. A significant positive correlation between both adipomyokines (METRNL and irisin) with r = 0.224 and *p* = 0.001 (Figure 5A). There was a significant positive correlation between METRNL and irisin among the T2D individuals (Figure 5B) with r = 0.364 and *p* ≤ 0.001. This was not seen within the non-diabetic population. Among all of the clinical parameters, a strong positive correlation was observed between METRNL level and BMI (r = 0.206, *p* = 0.002) and between METRNL level and HbA1c (r = 0.137, *p* = 0.044). On the other hand, irisin had a strong positive correlation with BMI, TC, LDL, TGL, FBG, and HbA1c. As shown in Table 4, a strong negative correlation was observed between irisin with HDL. To further understand the effect of T2D on the correlation of METRNL and irisin with different physical, clinical and biochemical parameters, we classified the population as non-diabetic and T2D, adjusting for age and gender. The results showed significant positive association of METRNL with BMI (r = 0.239, *p* = 0.015) and TC (r = 0.207, *p* = 0.039) in T2D individuals. We also observed, significant positive correlations between irisin and BMI, TC, LDL, TG, and FBG as shown in Table 5. Interestingly, a similar positive correlation was observed between irisin and TC, LDL and TG in the non-diabetic population. A strong negative correlation was observed between irisin and HDL in the non-diabetic population (Table 6).

## 4. Discussion

Our study shows that plasma levels of METRNL and irisin are increased in T2D patients as well as in obese individuals, which is further exacerbated in obese T2D individuals. Notably, for the first time our data revealed a strong positive correlation between METRNL and irisin in our population independent of age and gender. Interestingly, this association was more pronounced in the male population. This data is particularly interesting as both these adipomyokines are regulated by the transcription factor PGC-1α. Moreover, both proteins are involved in stimulating white adipose tissue browning, promoting glucose uptake in the skeletal muscle and heart, improving hepatic glucose and lipid metabolism, and enhancing pancreatic β-cell function [28,29,30]. Accordingly, an increased level of these proteins may be beneficial in improving insulin sensitivity. However, the increased plasma levels of METRNL and irisin in T2D and obese individuals remains paradoxical. Pardo et al. suggested that circulating irisin levels are affected by the amount secreted by the muscle and adipose tissues as well as by the physiological condition of the study subjects. For instance, skeletal muscle tissues release irisin in response to exercise, while the majority of irisin is released from the adipose tissue in obese individuals [31]. Similarly, this could possibly explain the higher levels of METRNL that are secreted by muscle and adipose tissues. Our findings are supported by several recent studies assessing circulation levels of either METRNL or irisin in T2D individuals, which found that the protein levels are higher in T2D patients [32,33,34,35,36]. The increased levels of irisin and METRNL in the circulation in the T2D and obese subjects may be due to a physiological attempt to restore glucose tolerance or a defense mechanism to counteract metabolic stress or resistance to these proteins, including insulin and leptin resistance [33,34,37].

In the present study, we observed a significant positive association between the plasma levels of METRNL with TC and BMI. A remarkable positive correlation was shown between the plasma levels of irisin and BMI, TC, LDL, TG, FBG and HbA1c. Previous studies have demonstrated that irisin and METRNL levels are positively associated with higher BMI and various obesity- and T2D-related parameters [35,36]. For example, a recent study found that serum irisin level was positively correlated with BMI, TC, TG, LDL, FBG, HbA1c, fasting insulin, and HOMA-IR, and showed a significant negative correlation with HDL [38]. This is in agreement with our results on the irisin level in circulation and its negative association with HDL. Knowing that HDL plays a protective role in cardiovascular diseases, inflammation and metabolism [39] The strong negative correlation we observe between irisin and HDL, may indicate that increased level of irisin may act as a pro-inflammatory molecule leading to reduced insulin sensitivity and dysregulation of metabolic pathways associated with obesity [38].

There are conflicting data regarding the levels of METRNL and irisin in the blood circulation and their association with various markers for T2D and obesity. In 2017, the International Medical Society conducted a meta-analysis that summarized studies performed between 2012 and 2016 focusing on circulating irisin levels in individuals with T2D and obesity. They concluded that individuals with newly diagnosed T2D have low circulation levels of irisin [40,41,42]. In addition, a recent study by Lee et al. showed that serum METRNL concentrations were significantly lower in patients with newly diagnosed T2D [43]. Al-Daghri et al. performed a single nucleotide polymorphism (SNP) analysis in a Saudi population and evaluated five SNPs of the FNDC5 (irisin) gene. They reported variations in the association of various SNPs with serum lipids, insulin, HOMA-IR, and irisin, which could be a result of the effect of SNPs on health-related phenotypes and which may not be consistent among diverse ethnic groups due to differences in genetic backgrounds and allelic frequencies [18]. This could explain the conflicting data regarding the level of these proteins in T2D and obesity.

In conclusion, our study confirms an increased expression level of both irisin and METRNL proteins in obesity and T2D. In addition, both proteins demonstrated a strong positive correlation with each other, suggesting a possible relationship between them. Further studies are needed to elucidate the role of these proteins, their interplay, and their mechanistic significance in the pathophysiology of T2D and obesity. Also to better understand their ethnic and gender specific variations.

## Figures and Tables

**Table 1:** Characteristics of non-diabetic and type 2 diabetic (T2D) study subjects.

Phenotype	Non-Diabetic (n = 123)	T2D (n = 104)	*p*-Value
Gender (M/F)	M (45); F (78)	M (55); F (49)	
Age (years)	41.80 ± 1.10	52.30 ± 0.91	<0.001
^†^ BMI (kg/m^2^)	28.86 ± 0.48	31.62 ± 0.42	<0.001
Waist/Hip ratio	0.86 ± 0.01	0.96 ± 0.02	<0.001
^‡^ TC (mmol/L)	5.09 ± 0.09	4.89 ± 0.13	0.207
^§^ HDL (mmol/L)	1.37 ± 0.04	1.18 ± 0.05	<0.001
^‖^ LDL (mmol/L)	3.20 ± 0.08	3.00 ± 0.11	0.155
^¶^ TG (mmol/L)	1.16 ± 0.08	1.63 ± 0.11	<0.001
^††^ FBG (mg/L)	95.76 ± 1.8	146.88 ± 5.4	<0.001
^‡‡^ HbA1c (DCCT%)	5.56 ± 0.06	7.68 ± 0.18	<0.001
Insulin (mU/L)	9.15 ± 0.61	15.17 ± 1.19	<0.001
C-peptide (μg/L)	3.89 ± 0.35	2.76 ± 0.24	0.008
^§§^ HOMA-IR (AU)	0.53 ± 0.04	0.86 ± 0.06	<0.001
^¶¶^ HOMA-β (AU)	28.49 ± 1.89	14.82 ± 1.19	<0.001

*p*-value was calculated using the Mann–Whitney *U* test. Data are presented as mean ± Standard Error of Mean (SEM). The Mann–Whitney *U* test was used to compare various clinical and biochemical parameters (n = 228): ^†^ BMI (body mass index); ^‡^ TC (total cholesterol); ^§^ HDL (high-density lipoprotein); ^‖^ LDL (low-density lipoprotein); ^¶^ TG (triglyceride level); ^††^ FBG (fasting blood glucose); ^‡‡^ HbA_1c_ (hemoglobin A1c); ^§§^ homeostatic model assessment (HOMA) for insulin resistance (HOMA-IR) and for ^¶¶^ β-cell function (HOMA-β).

**Table 2:** Characteristics of non-obese and obese study subjects.

Phenotype	Non-Obese (n = 111)	Obese (n = 117)	*p*-Value
Gender (M/F)	M (47); F (64)	M (53); F (63)	
Age (years)	44.20 ± 1.17	48.91 ± 1.07	<0.001
^†^ BMI (kg/m^2^)	25.86 ± 0.27	34.21 ± 0.26	<0.001
Waist/Hip ratio	0.86 ± 0.01	0.94 ± 0.02	<0.001
^‡^ TC (mmol/L)	5.01 ± 0.12	5.00 ± 0.10	0.927
^§^ HDL (mmol/L)	1.34 ± 0.05	1.23 ± 0.04	0.077
^‖^ LDL (mmol/L)	3.13 ± 0.10	3.10 ± 0.09	0.754
^¶^ TG (mmol/L)	1.25 ± 0.10	1.49 ± 0.10	0.081
^††^ FBG (mg/L)	106.38 ± 3.42	131.40 ± 5.04	<0.001
^‡‡^ HbA1c (DCCT%)	5.95 ± 0.11	7.09 ± 0.18	<0.001
Insulin (mU/L)	11.78 ± 1.01	12.57 ± 1.00	0.577
C-peptide (μg/L)	3.22 ± 0.32	3.45 ± 0.30	0.594
^§§^ HOMA-IR (AU)	0.58 ± 0.27	0.79 ± 0.05	0.002
^¶¶^ HOMA-β (AU)	22.83 ± 1.53	20.74 ± 1.86	0.386

*p*-value was calculated using Mann–Whitney *U* test Data are presented as mean ± Standard Error of Mean (SEM). Mann–Whitney *U* test was used to compare various clinical and biochemical parameters (n = 228): ^†^ BMI (body mass index); ^‡^ TC (total cholesterol); ^§^ HDL (high-density lipoprotein); ^‖^ LDL (low-density lipoprotein); ^¶^ TG (triglyceride level); ^††^ FBG (fasting blood glucose); ^‡‡^ HbA1c (hemoglobin A1c); ^§§^ homeostatic model assessment (HOMA) for insulin resistance (HOMA-IR) and for ^¶¶^ β-cell function (HOMA-β).

**Table 3:** Physical and biochemical characteristics of the non-diabetic and diabetic population categorized based on obesity.

Phenotype	Non-Diabetic (n = 124)			Diabetic (n = 104)		
	Non-Obese (n = 73)	Obese (n = 51)	*p*-Value	Non-Obese (n = 38)	Obese (n = 66)	*p*-Value
Gender (M/F)	M (26); F (47)	M (19); F (31)		M (21); F (17)	M (34); F (32)	
Age (years)	40.5 ± 1.40	43.64 ± 1.77	0.171	51.24 ± 1.60	52.91 ± 1.11	0.392
^†^ BMI (kg/m^2^)	25.31 ± 0.35	34.04 ± 0.45	<0.001	26.89 ± 0.37	34.34 ± 0.30	<0.001
Waist/Hip ratio	0.83 ± 0.02	0.89 ± 0.01	0.005	0.92 ± 0.02	0.98 ± 0.03	0.072
^‡^ TC (mmol/L)	5.10 ± 0.11	5.09 ± 0.14	0.939	4.84 ± 0.27	4.93 ± 0.14	0.769
^§^ HDL (mmol/L)	1.39 ± 0.05	1.34 ± 0.05	0.478	1.23 ± 0.10	1.15 ± 0.05	0.435
^‖^ LDL (mmol/L)	3.19 ± 0.1	3.21 ± 0.13	0.897	3.02 ± 0.21	3.0 ± 0.13	0.918
^¶^ TG (mmol/L)	1.12 ± 0.12	1.214 ± 0.10	0.532	1.50 ± 0.18	1.71 ± 0.15	0.373
^††^ FBG (mg/L)	94.14 ± 2.7	98.1 ± 2.34	0.267	129.78 ± 6.84	156.6 ± 7.38	0.009
^‡‡^ HbA1c (DCCT %)	5.56 ± 0.09	5.57 ± 0.08	0.892	6.66 ± 0.21	8.23 ± 0.22	<0.001
Insulin (mU/L)	8.96 ± 0.79	9.58 ± 0.91	0.609	16.80 ± 2.16	14.13 ± 1.37	0.3
C-peptide (μg/L)	3.65 ± 0.43	4.22 ± 0.59	0.438	2.48 ± 0.40	2.91 ± 0.29	0.38
^§§^ HOMA-IR (AU)	5.10 ± 1.53	3.91 ± 0.92	0.51	0.79 ± 0.11	0.89 ± 0.06	0.448
^¶¶^ HOMA-β (AU)	0.44 ± 0.03	0.65 ± 0.07	0.008	15.90 ± 1.88	14.24 ± 1.53	0.495

Data are presented as mean ± Standard Error of Mean (SEM). One-way ANOVA and post-hoc Bonferroni tests were used to compare various clinical and biochemical parameters (n = 228): ^†^ BMI (body mass index); ^‡^ TC (total cholesterol); ^§^ HDL (high-density lipoprotein); ^‖^ LDL (low-density lipoprotein); ^¶^ TG (triglyceride level); ^††^ FBG (fasting blood glucose); ^‡‡^ HbA1c (hemoglobin A1c); ^§§^ homeostatic model assessment (HOMA) for insulin resistance (HOMA-IR) and for ^¶¶^ β-cell function (HOMA-β).

**Table 4:** Correlation between circulating METRNL and irisin proteins and physical, clinical, and biochemical parameters in all population adjusted for age and gender.

Phenotype (All Populations)	METRNL		Irisin	
	r^2^	*p*-Value	r^2^	*p*-Value
^†^ BMI (kg/m^2^)	0.206 **	0.002	0.245 ***	<0.001
Waist/Hip ratio	0.113	0.168	0.052	0.546
^‡^ TC (mmol/L)	0.040	0.556	0.260 ***	<0.001
^§^ HDL (mmol/L)	−0.110	0.103	−0.196 **	0.004
^‖^ LDL (mmol/L)	0.087	0.197	0.240 ***	<0.001
^¶^ TG (mmol/L)	0.083	0.221	0.377 ***	<0.001
^††^ FBG (mg/L)	0.128	0.058	0.224 **	0.001
^‡‡^ HbA1c (DCCT %)	0.137 *	0.044	0.256 ***	<0.001
Insulin (mU/L)	0.118	0.142	0.145	0.058
C-peptide (μg/L)	0.056	0.503	−0.064	0.476
^§§^ HOMA-IR (AU)	−0.024	0.750	0.127	0.086
^¶¶^ HOMA-β (AU)	−0.096	0.199	−0.117	0.113

Levels of METRNL and irisin obtained using ELISA and various physical and clinical parameters: ^†^ BMI (body mass index); ^‡^ TC (total cholesterol); ^§^ HDL (high-density lipoprotein); ^‖^ LDL (low-density lipoprotein); ^¶^ TG (triglyceride level); ^††^ FBG (fasting blood glucose); ^‡‡^ HbA1c (hemoglobin A1c); ^§§^ homeostatic model assessment (HOMA) for insulin resistance (HOMA-IR) and for ^¶¶^ β-cell function (HOMA-β). Correlations statistically significant at * *p* < 0.05; ** *p* < 0.01; *** *p* < 0.001.

**Table 5:** Correlation between circulating METRNL and irisin proteins and physical, clinical, and biochemical parameters in T2D population adjusted for age and gender.

Phenotype (T2D)	METRNL		Irisin	
	r^2^	*p*-Value	r^2^	*p*-Value
^†^ BMI (kg/m^2^)	0.239 *	0.015	0.292 **	0.005
Waist/Hip ratio	0.119	0.317	0.057	0.642
^‡^ TC (mmol/L)	0.207 *	0.039	0.321 **	0.002
^§^ HDL (mmol/L)	−0.013	0.896	−0.114	0.289
^‖^ LDL (mmol/L)	0.189	0.062	0.238 *	0.025
^¶^ TG (mmol/L)	0.133	0.188	0.416 ***	<0.001
^††^ FBG (mg/L)	0.083	0.407	0.213 *	0.043
^‡‡^ HbA1c (DCCT%)	0.126	0.210	0.202	0.056
Insulin (mU/L)	0.067	0.565	0.150	0.189
C-peptide (μg/L)	0.173	0.180	0.048	0.738
^§§^ HOMA-IR (AU)	−0.073	0.494	−0.025	0.815
^¶¶^ HOMA-β (AU)	−0.120	0.262	−0.231 *	0.029

Meteorin-like hormone (METRNL). Spearman rank correlation was assessed based on plasma levels of METRNL and irisin obtained using ELISA and various physical and clinical parameters: ^†^ BMI (body mass index); ^‡^ TC (total cholesterol); ^§^ HDL (high-density lipoprotein); ^‖^ LDL (low-density lipoprotein); ^¶^ TG (triglyceride level); ^††^ FBG (fasting blood glucose); ^‡‡^ HbA1c (hemoglobin A1c); ^§§^ homeostatic model assessment (HOMA) for insulin resistance (HOMA-IR) and for ^¶¶^ β-cell function (HOMA-β). Correlations statistically significant at * *p* < 0.05; ** *p* < 0.01; *** *p* < 0.001.

**Table 6:** Correlation between circulating METRNL and irisin proteins and physical, clinical, and biochemical parameters in non-diabetic population adjusted for age and gender.

Phenotype (Non-Diabetic)	METRNL		Irisin	
	r^2^	*p*-Value	r^2^	*p*-Value
^†^ BMI (kg/m^2^)	0.120	0.188	0.132	0.146
Waist/Hip ratio	−0.001	0.992	−0.120	0.327
^‡^ TC (mmol/L)	−0.078	0.400	0.243 **	0.007
^§^ HDL (mmol/L)	−0.115	0.212	−0.211 *	0.020
^‖^ LDL (mmol/L)	0.017	0.852	0.262 **	0.004
^¶^ TG (mmol/L)	−0.041	0.659	0.289 **	0.001
^††^ FBG (mg/L)	−0.059	0.522	0.039	0.674
^‡‡^ HbA1c (DCCT%)	0.019	0.842	0.226 *	0.015
Insulin (mU/L)	0.072	0.534	0.044	0.676
C-peptide (μg/L)	−0.051	0.652	−0.222	0.059
^§§^ HOMA-IR (AU)	−0.102	0.340	0.155	0.138
^¶¶^ HOMA-β (AU)	−0.030	0.778	0.177	0.089

Meteorin-like hormone (METRNL). Spearman rank correlation was assessed based on plasma levels of METRNL and irisin obtained using ELISA and various physical and clinical parameters: ^†^ BMI (body mass index); ^‡^ TC (total cholesterol); ^§^ HDL (high-density lipoprotein); ^‖^ LDL (low-density lipoprotein); ^¶^ TG (triglyceride level); ^††^ FBG (fasting blood glucose); ^‡‡^ HbA1c (hemoglobin A1c); ^§§^ homeostatic model assessment (HOMA) for insulin resistance (HOMA-IR) and for ^¶¶^ β-cell function (HOMA-β). Correlations statistically significant at * *p* < 0.05; ** *p* < 0.01.

## Supplementary Materials

The following are available online at https://www.mdpi.com/2073-4409/8/10/1283/s1. Supplementary Figure S1: METRNL level in plasma in all populations (n = 228). Supplementary Figure S2: METRNL and irisin level in plasma in all population (n = 228) categorized by age. Supplementary Figure S3: Irisin level in plasma in all populations (n = 228). Supplementary Figure S4: Correlation analysis between METRNL and irisin levels in plasma in the population (n = 228) categorized based on gender. Supplementary Table S1: Correlation between circulating METRNL and irisin proteins and physical, clinical, and biochemical parameters in lean population adjusted for age and gender. Supplementary Table S2: Correlation between circulating METRNL and irisin proteins and physical, clinical, and biochemical parameters in overweight population adjusted for age and gender. Supplementary Table S3: Correlation between circulating METRNL and irisin proteins and physical, clinical, and biochemical parameters in obese population adjusted for age and gender. Supplementary Table S4: Correlation between circulating METRNL and irisin proteins and physical, clinical, and biochemical parameters in male population adjusted for age. Supplementary Table S5: Correlation between circulating METRNL and irisin proteins and physical, clinical, and biochemical parameters in female population adjusted for age.
