# Supplementary material for: Increased Expression of Meteorin-Like Hormone in Type 2 Diabetes and Obesity and Its Association with Irisin
Source: Cells. 2019 Oct 19;8(10):1283. doi: 10.3390/cells8101283 (PMC6829873; doi:10.3390/cells8101283)
Supplement: Supplementary file 1 [file cells-08-01283-s001.pdf]

cells

MDPI

Supplementary Data

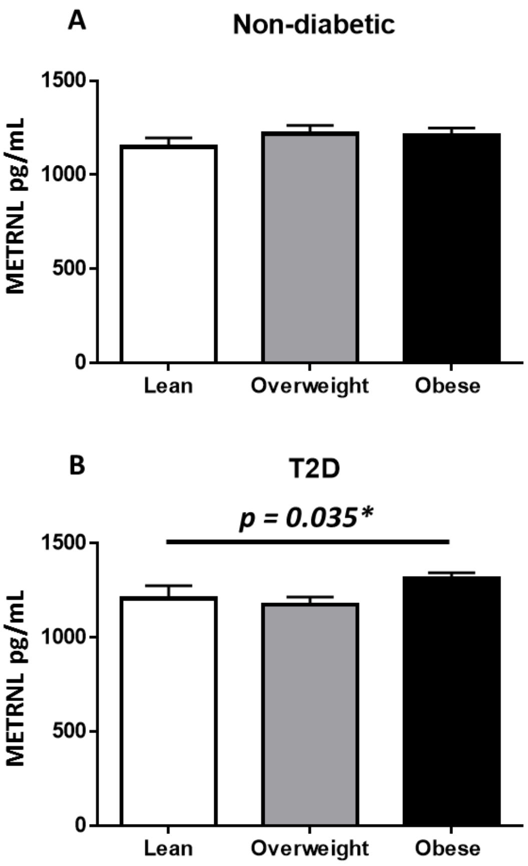

Supplementary Figure S1: METRNL level in plasma in all populations (n=228). (A) Comparing lean, overweight and obese in non-diabetic individuals; **(B)** Comparing lean, overweight and obese in T2D individuals. METRNL level in plasma was determined using Enzyme linked Immunosorbent Assay. The population was classified on the basis of their diabetic status. Diabetes was defined by fasting plasma glucose ≥ 126mg/L (7 mmol/L). Furthermore, the population was classified on the basis of Obesity. Obesity was defined based on BMI, where participants with BMI > 30 kg/m^2 were considered obese, those with BMI between 25 and 30 kg/m^2 were considered overweight and participants with BMI < 25 kg/m^2 were considered lean. Statistical assessment was 2-sided and considered statistically significant at *$p < 0.05$.

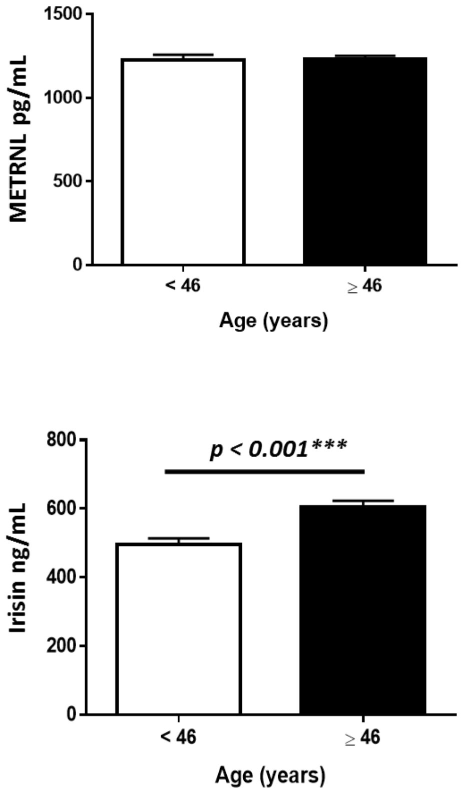

Supplementary Figure S2: METRNL and irisin level in plasma in all population (n=228) categorized by age. (A) Comparing the plasma level of METRNL with increasing age. **(B)** Comparing the plasma level of irisin with increasing age. METRNL and irisin level in plasma was determined using Enzyme linked Immunosorbent Assay. The population was classified on the basis of their mean age (46 years). Statistical assessment was 2-sided and considered statistically significant at ***$p < 0.001$.

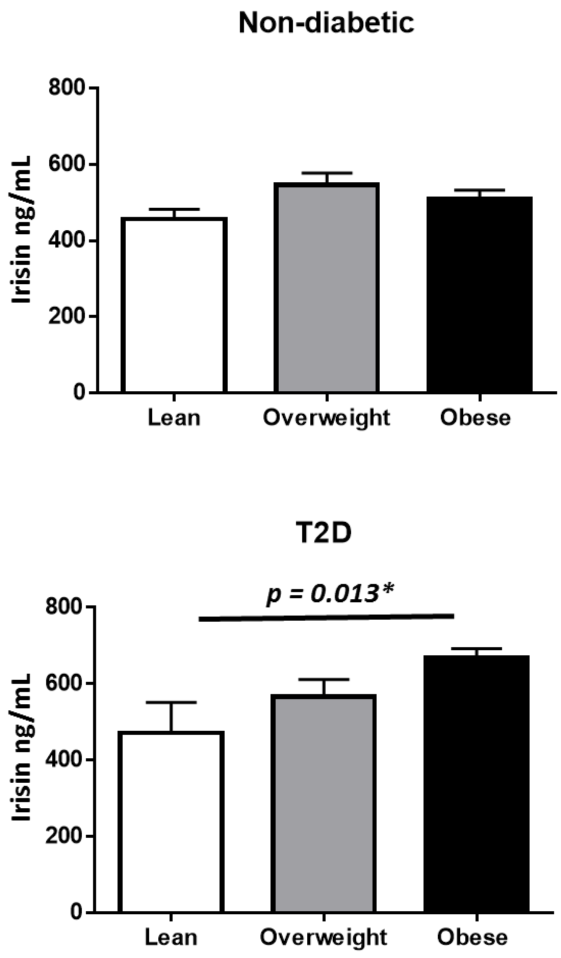

Supplementary Figure S3: Irisin level in plasma in all populations (n=228). (A) Comparing lean, overweight and obese in non-diabetic individuals; **(B)** Comparing lean, overweight and obese in T2D individuals. Irisin level in plasma was determined using Enzyme linked Immunosorbent Assay. The population was classified on the basis of their diabetic status. Diabetes was defined by fasting plasma glucose ≥ 126mg/L (7 mmol/L). Furthermore, the population was classified on the basis of Obesity. Obesity was defined based on BMI, where participants with BMI > 30 kg/m^2 were considered obese, those with BMI between 25 and 30 kg/m^2 were considered overweight and participants with BMI < 25 kg/m^2 were considered lean. Statistical assessment was 2-sided and considered statistically significant at $*p < 0.05$.

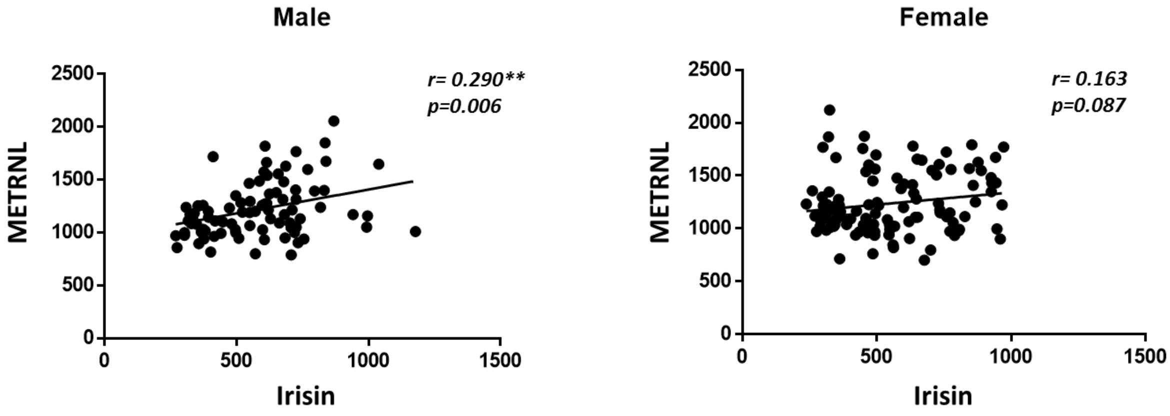

Supplementary Figure S4: Correlation analysis between METRNL and irisin levels in plasma in the population (n=228) categorized based on gender. (A) Correlation in Male population. **(B)** Correlation in female population. METRNL and irisin levels in plasma were determined using Enzyme linked Immunosorbent Assay. Spearman correlation coefficient was used to determine the association of METRNL with irisin. Statistical assessment was 2-sided and considered statistically significant at ***$p < 0.01$.

Supplementary Table S1: Correlation between circulating METRNL and irisin proteins and physical, clinical, and biochemical parameters in lean population adjusted for age and gender.

Phenotype (Lean) n=35	METRNL		Irisin	
	r^2	*P-value*	r^2	*P-value*
†BMI (kg/m^2)	0.276	0.103	0.122	0.471
Waist/Hip ratio	0.455	0.102	0.418	0.155
‡TC (mmol/L)	0.044	0.804	0.209	0.228
§HDL (mmol/L)	-0.114	0.521	-0.060	0.732
‖ LDL (mmol/L)	0.181	0.306	0.073	0.677
¶TG (mmol/L)	-0.021	0.908	0.244	0.165
††FBG (mg/L)	0.025	0.889	0.273	0.118
‡‡HbA1c (DCCT %)	-0.089	0.629	0.178	0.322
Insulin (mU/L)	-0.016	0.931	-0.288	0.084
C-peptide (µg/L)	-0.059	0.756	-0.385*	**0.036**
§§HOMA-IR (AU)	-0.114	0.587	0.092	0.670
¶¶HOMA-β (AU)	-0.308	0.134	-0.155	0.468

Meteorin-like hormone (METRNL). Spearman rank correlation was assessed based on plasma levels of METRNL and irisin obtained using ELISA and various physical and clinical parameters: †BMI (body mass index); ‡TC (total cholesterol); §HDL (high-density lipoprotein); ‖ LDL (low-density lipoprotein); ¶TG (triglyceride level); ††FBG (fasting blood glucose); ‡‡HbA1c (hemoglobin A1c); §§homeostatic model assessment (HOMA) for insulin resistance (HOMA-IR) and for ¶¶β-cell function (HOMA-β). Correlations statistically significant at *$p < 0.05$.

Supplementary Table S2: Correlation between circulating METRNL and irisin proteins and physical, clinical, and biochemical parameters in overweight population adjusted for age and gender.

**Phenotype (Overweight) n=69**	**METRNL**		**Irisin**	
	**r^2**	***P-value***	**r^2**	***P-value***
**†BMI (kg/m²)**	0.181	0.132	0.271*	**0.028**
**Waist/Hip ratio**	-0.220	0.121	-0.094	0.521
**‡TC (mmol/L)**	0.047	0.697	0.254*	**0.041**
**§HDL (mmol/L)**	-0.021	0.861	-0.255	0.040
**‖ LDL (mmol/L)**	0.077	0.527	0.242	0.052
**¶TG (mmol/L)**	0.010	0.932	0.382**	**0.002**
**††FBG (mg/L)**	0.004	0.973	0.134	0.287
**‡‡HbA1c (DCCT %)**	-0.115	0.356	-0.035	0.789

**Insulin (mU/L)**	0.199	0.170	0.248	0.073
**C-peptide (μg/L)**	0.026	0.864	0.093	0.568
**§§HOMA-IR (AU)**	-0.214	0.117	0.248	0.063
**¶¶HOMA-β (AU)**	-0.092	0.503	-0.036	0.791

Meteorin-like hormone (METRNL). Spearman rank correlation was assessed based on plasma levels of METRNL and irisin obtained using ELISA and various physical and clinical parameters: †BMI (body mass index); ‡TC (total cholesterol); §HDL (high-density lipoprotein); ‖ LDL (low-density lipoprotein); ¶TG (triglyceride level); ††FBG (fasting blood glucose); ‡‡HbA1c (hemoglobin A1c); §§homeostatic model assessment (HOMA) for insulin resistance (HOMA-IR) and for ¶¶β-cell function (HOMA-β). Correlations statistically significant at $*p < 0.05$; $**p < 0.01$.

Supplementary Table S3: Correlation between circulating METRNL and irisin proteins and physical, clinical, and biochemical parameters in obese population adjusted for age and gender.

**Phenotype (Obese) n=113**	**METRNL**		**Irisin**	
	**r^2**	***P-value***	**r^2**	***P-value***
**†BMI (kg/m^2)**	0.118	0.210	0.137	0.154
**Waist/Hip ratio**	0.133	0.232	0.032	0.791
**‡TC (mmol/L)**	0.021	0.822	0.285**	**0.003**
**§HDL (mmol/L)**	-0.132	0.163	-0.193*	**0.046**

**‖ LDL (mmol/L)**	0.065	0.498	0.261**	**0.007**
**¶TG (mmol/L)**	0.080	0.397	0.367***	**<0.001**
**††FBG (mg/L)**	0.193*	**0.039**	0.207*	**0.030**
**‡‡HbA1c (DCCT %)**	0.240*	**0.010**	0.345***	**<0.001**
**Insulin (mU/L)**	0.063	0.602	0.241*	**0.032**
**C-peptide (µg/L)**	0.102	0.428	0.082	0.560
**§§HOMA-IR (AU)**	-0.044	0.670	-0.024	0.813
**¶¶HOMA-β (AU)**	-0.119	0.246	-0.183	0.071

Meteorin-like hormone (METRNL). Spearman rank correlation was assessed based on plasma levels of METRNL and irisin obtained using ELISA and various physical and clinical parameters: †BMI (body mass index); ‡TC (total cholesterol); §HDL (high-density lipoprotein); ‖ LDL (low-density lipoprotein); ¶TG (triglyceride level); ††FBG (fasting blood glucose); ‡‡HbA1c (hemoglobin A1c); §§homeostatic model assessment (HOMA) for insulin resistance (HOMA-IR) and for ¶¶β-cell function (HOMA-β). Correlations statistically significant at *$p < 0.05$; **$p < 0.01$; ***$p < 0.001$.

Supplementary Table S4: Correlation between circulating METRNL and irisin proteins and physical, clinical, and biochemical parameters in male population adjusted for age.

**Phenotype (Male population) n=98**	**METRNL**		**Irisin**	
	**r^2**	***P-value***	**r^2**	***P-value***

**†BMI (kg/m^2)**	0.396***	**<0.001**	0.232*	**0.021**
**Waist/Hip ratio**	0.273*	**0.026**	0.368**	**0.004**
**‡TC (mmol/L)**	0.080	0.434	0.272**	**0.008**
**§HDL (mmol/L)**	-0.157	0.128	-0.169	0.103
**‖ LDL (mmol/L)**	0.059	0.569	0.176	0.092
**¶TG (mmol/L)**	0.210*	**0.039**	0.457***	**<0.001**
**††FBG (mg/L)**	0.223*	**0.027**	0.284**	**0.005**
**‡‡HbA1c (DCCT %)**	0.237*	**0.021**	0.293**	**0.004**
**Insulin (mU/L)**	0.182	0.138	0.278*	**0.014**
**C-peptide (µg/L)**	0.088	0.502	0.038	0.786
**§§HOMA-IR (AU)**	-0.016	0.893	0.184	0.107
**¶¶HOMA-β (AU)**	-0.181	0.118	-0.206	0.071

Meteorin-like hormone (METRNL). Spearman rank correlation was assessed based on plasma levels of METRNL and irisin obtained using ELISA and various physical and clinical parameters: †BMI (body mass index); ‡TC (total cholesterol); §HDL (high-density lipoprotein); ‖ LDL (low-density lipoprotein); ¶TG (triglyceride level); ††FBG (fasting blood glucose); ‡‡HbA1c (hemoglobin A1c); §§homeostatic model assessment (HOMA) for insulin resistance (HOMA-IR) and for ¶¶β-cell function (HOMA-β). Correlations statistically significant at $*p < 0.05$; $**p < 0.01$; $***p < 0.001$.

Supplementary Table S5: Correlation between circulating METRNL and irisin proteins and physical, clinical, and biochemical parameters in female population adjusted for age.

Phenotype (Female population) n=124	METRNL		Irisin	
	r^2	*P-value*	r^2	*P-value*
[†]BMI (kg/m^2)	0.111	0.214	0.256**	**0.005**
Waist/Hip ratio	-0.019	0.860	-0.116	0.313
[‡]TC (mmol/L)	0.017	0.847	0.275**	**0.003**
[§]HDL (mmol/L)	-0.113	0.208	-0.200*	**0.030**
[‖] LDL (mmol/L)	0.126	0.163	0.303**	**0.001**
[¶]TG (mmol/L)	0.020	0.823	0.327***	**<0.001**
[††]FBG (mg/L)	0.052	0.563	0.172	0.063
[‡‡]HbA1c (DCCT %)	0.069	0.451	0.226*	**0.015**
Insulin (mU/L)	0.044	0.682	0.047	0.649
C-peptide (µg/L)	0.037	0.740	-0.102	0.390
[§§]HOMA-IR (AU)	-0.087	0.380	0.097	0.324
[¶¶]HOMA-β (AU)	-0.070	0.479	-0.065	0.510

Meteorin-like hormone (METRNL). Spearman rank correlation was assessed based on plasma levels of METRNL and irisin obtained using ELISA and various physical and clinical parameters: †BMI (body mass index); ‡TC (total cholesterol); §HDL (high-density lipoprotein); ‖ LDL (low-density lipoprotein); ¶TG (triglyceride level); ††FBG (fasting blood glucose); ‡‡HbA1c (hemoglobin A1c); §§homeostatic model assessment (HOMA) for insulin resistance (HOMA-IR) and for ¶¶β-cell function (HOMA-β). Correlations statistically significant at *$p < 0.05$; **$p < 0.01$; ***$p < 0.001$.
